# Experiences of and attitudes to lifestyle modification for the management of multiple sclerosis: A qualitative analysis of free‐text survey data

**DOI:** 10.1111/hex.13364

**Published:** 2021-10-02

**Authors:** Sandra L. Neate, Angela Donald, George A. Jelinek, Nupur Nag

**Affiliations:** ^1^ Neuroepidemiology Unit, Centre for Epidemiology and Biostatistics, Melbourne School of Population and Global Health The University of Melbourne Parkville Victoria Australia

**Keywords:** barriers, challenges, enablers, lifestyle modification, multiple sclerosis, MS management, outcomes

## Abstract

**Background:**

Growing evidence suggests a role of lifestyle modification in improved health outcomes for people with multiple sclerosis (pwMS); however, perspectives of pwMS who engage in lifestyle modification are lacking.

**Objective:**

We explored perspectives of pwMS regarding the modification of lifestyle‐related risk factors in multiple sclerosis (MS) for disease management to understand attitudes to and experiences of lifestyle modification as part of self‐management from a patient perspective.

**Design:**

Participants were ≥18 years and English speaking who responded to a free‐text open‐ended question in the Health Outcomes and Lifestyle In a Sample of pwMS (HOLISM), an international online survey. Responses were analysed utilizing inductive thematic analysis.

**Results:**

Under the exploration of lifestyle modification, themes describing the experiences and attitudes of participants included practical challenges and physical and psychological barriers, enablers of change and experienced outcomes. Although participants reported some practical and psychological challenges to adoption and maintenance of lifestyle behaviours, many expressed an ability to gain control of MS through engagement with lifestyle behaviours and the development of hope and optimism that accompanied this sense of control, at times leading to a sense of personal transformation.

**Conclusion:**

Findings highlight the challenges experienced by pwMS in adopting lifestyle modifications for disease management as well as the positive benefits from following healthy lifestyle behaviours. Our findings may form the basis of more focussed qualitative explorations of the experiences and outcomes of lifestyle modification in MS in the future.

**Patient Contribution:**

Consenting pwMS completed a survey capturing data on demographics, clinical course, lifestyle behaviours and health outcomes.

## 1 INTRODUCTION

Multiple sclerosis (MS) is an inflammatory‐neurodegenerative disease of the central nervous system characterized by demyelination and axonal degeneration, which manifests in a heterogeneous array of physical and psychological symptoms.^1^ Up to 85% of people with MS (pwMS) experience relapsing remitting MS (RRMS), which is frequently preceded by a first acute clinical attack known as ‘clinically isolated syndrome’.^1^ Over time, neurodegenerative axonal injury and atrophy lead to the development of a secondary progressive course in 80% of those with RRMS.^2^ Regardless of disease phenotype, the potential for disability, including impairments in vision and mobility, loss of balance, bladder and bowel disturbances, is of major concern to all pwMS.

As a result of both the limited efficacy of pharmaceutical management and the experiences of deterioration in health for pwMS, the demand for nonpharmaceutical interventions has gained importance for pwMS.^3^, ^4^ PwMS are often proactive in their efforts to remain healthy and frequently seek advice from clinicians regarding factors that may slow or prevent disease progression.^5^ There is growing evidence that lifestyle modification, which is the altering of long‐term habits, commonly diet and physical activity, and adoption and maintenance of behaviours aimed at the reduction of recognized risk factors, is an effective means to manage disease progression and symptom severity in MS.^6^ Abstinence from smoking, a healthy diet, physical activity, adequate vitamin D levels, supplementation with omega 3 fatty acids and meditation are all associated with a reduction in the chronic inflammatory state associated with MS^7^ and subsequent improved health outcomes. These outcomes include better mental and physical health quality of life,^8^, ^9^, ^10^ reduced relapse rate,^11^ depression risk^12^ and clinically significant fatigue^13^ and lower burden in terms of disability and symptoms.^6^, ^14^

Due to many factors, including the unpredictable manifestation and progression of MS, the frequently early age of onset and the preponderance of the disease in women of childbearing age, even apparently straightforward decisions, such as taking medications, are experienced as not simply individual and rational decisions, but complex matters that occur in the context of the person's life as a whole and ‘a web of relationships with relatives and friends’.^15^ It is therefore understandable that adopting lifestyle modification may present many complexities and challenges to the individual. However, there is a paucity of information regarding what drives and impedes motivation, the facilitators and barriers to implementation and the personal perspectives of what lifestyle modification has meant to those adopting change.

Though pwMS often adopt lifestyle behaviours for disease self‐management, few studies have examined their perspectives and experiences of making these changes. In previous qualitative studies of pwMS who adopt lifestyle change, nutrition and other lifestyle factors (stress, sleep and temperature) have been perceived to affect disease activity, even on a daily basis.^16^ Barriers and enablers to exercise adoption, the perceived value of exercise in sustaining independence and the need for clear guidance from health professionals about the optimum mode and amount of exercise have been described.^17^ Studies of physical activity interventions in MS have reported the experience of increased self‐confidence and competence, as participants successfully pushed their perceived physical exercise boundaries, which reinforced their exercise capabilities.^18^, ^19^ Further, physical exercise interventions were powerful in teaching pwMS the skills and knowledge required to enact positive control over their behaviours, leading to feelings of mastery and overall wellness^18^, ^19^ and increased motivation to continue physical activity.^20^

This study analyses comments that specifically referred to the impact of engagement with lifestyle modification from an online international survey's concluding free‐text question. Our study aims to explore the experiences and attitudes of people engaging with lifestyle modification as part of MS management, and whether these experiences may be novel compared with the current limited attitudes and experiences described within the literature. In doing so, this study contributes towards enhancing MS patient‐centred care by providing clinicians with patient perspectives of lifestyle modifications and their utility in the management of MS.

## 2 MATERIALS AND METHODS

A qualitative descriptive study of free‐text responses to an open‐ended survey question was performed.

### 2.1 The survey

In 2012, participants were recruited via international promotion of the Health Outcomes and Lifestyle In a Sample of people with Ms (HOLISM) study on social media platforms and websites, the methodology of which has been previously described.^21^ Study participants were invited to complete an online 163 question survey, developed by researchers specifically for this project, capturing data on demographics, clinical course, lifestyle behaviours and health outcomes using validated tools, where available.

### 2.2 Participants

Participants were adults aged ≥18 years, English speaking and with a self‐reported clinician‐confirmed diagnosis of MS. Of the 2466 participants from 57 countries who completed the survey, 1006 (41%) responded to the open‐ended survey question ‘If there is anything else you would like to share with us, please write additional comments here’. Of these responders, 966 (96%) had a clinical diagnosis of MS and were included in the analysis.

### 2.3 Data collection and management

Deidentified responses were extracted from a secure database into Microsoft Excel and then imported into NVivo qualitative data analysis software (QSR International Pty Ltd. Version 12, 2018). A total of 514 participants' comments referred to lifestyle modification and constituted the final data set. Ethics approval ID: 1545102.

### 2.4 Qualitative analysis

Inductive thematic analysis was applied.^22^ This approach, whereby coding and theme development are derived from the raw data,^22^ is useful to express the complexities of meaning interlaced within textual data,^23^ and is consistent with other studies of free‐text survey data.^24^ Additionally, thematic analysis is beneficial when summarizing the key characteristics of a large data set, as researchers must be methodical in their handling of the data.^25^

**Figure 1:**
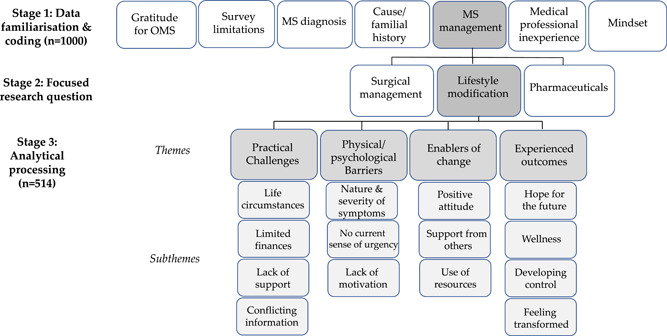
Schematic of the qualitative analysis process

The first stage of analysis was data familiarization (N. N. and A. D.). Comments were read several times, to gain a foundational understanding of the quality and depth of comments, followed by initial coding of data (N. N. and A. D.), to describe the nature of the comments. The second stage involved categorizing the codes through further reflection and analytical processing^26^ (N. N., S. L. N. and A. D.). It was during this process that the researchers decided to focus on and explore the comments relating to lifestyle modification, due to the richness of the data and the perceived interest and novelty of this exploration. Themes and subthemes that illustrated concepts within lifestyle modification were then derived from the data through a thoroughly iterative process during frequent researcher meetings. The analytical process is shown in Figure 1.

The research analysis team consisted of A. D. (Masters of Public Health student), N. N. (Senior Research Fellow) and S. L. N. (Senior Research Fellow). N. N. and S. L. N. have previous experience in conducting and publishing qualitative analyses.

The credibility of the analysis was enhanced by researcher immersion through multiple readings of the raw data; multiple researchers being involved in the interpretation; and through researcher reflective discussions.^27^ Frequent review of the data and initial coding and resulting codes, categories and themes ensured that the resultant themes accurately reflected the raw data and honoured the perspectives of the participants, enhancing the trustworthiness of the analysis.^25^ Detailed notes of researcher meetings were maintained by A. D. To enhance transparency, verbatim quotes have been used to illustrate themes, enabling readers to assess the validity and ‘fittingness’ of researcher interpretations.^28^

## 3 RESULTS

### 3.1 Themes

Four themes were identified during the exploration of lifestyle modification. These were practical challenges, physical and psychological barriers, enablers of change and experienced outcomes. With respect to participants' quotations, ‘Overcoming MS (OMS)’ is a lifestyle modification programme, and ‘Overcoming Multiple Sclerosis The evidence‐based seven step recovery program’ is the book to which some participants refer.^29^ The ‘OMS retreat/retreat’ is a residential lifestyle modification workshop. The ‘website’ is the OMS Charity website.

### 3.2 Practical challenges

Practical challenges included the multiple life‐related challenges for pwMS that hampered the initiation or maintenance of lifestyle modifications. The theme of practical challenges included four subthemes, that is, life circumstances, limited finances, conflicting information and lack of support.

Many expressed a desire to change aspects of their life; however, due to life circumstances and competing demands, such as lack of time and parental duties, this desire could not be initiated.

I'm a firm believer in lifestyle changes and dietary changes however I have found it hard to implement. A bit contradictory I know! With three young kids I do seem to still put myself last despite my illness … there is never enough time in the day!!

I had a baby a year ago. before that, I had been following Swank diet very well, with very good results (and no longer on MS medication). During pregnancy, I pretty much quit following the diet, and haven't been able to get back into.

Similarly, participants faced other practical challenges; for example, those who were unable to drive themselves found this to be a significant barrier when trying to increase their exercise levels. The lack of access to transport often meant that participants were unable to attend social events or gym classes, hindering activity that they longed to be a part of.

I am currently working to improve my diet and increase my exercise. I don't drive which makes it difficult to get to the gym or other social activities.

Further challenges included limited finances and a lack of support from family, friends or support services, leading to limitations to independence and ability to adopt recommended lifestyle behaviours.

I know my diet, exercise and such is poor, lack of funds and understanding in my family prevents changes at times. I do not shop and depend upon others to go places and my fatigue is severe.

Participants described the amount of conflicting information available particularly regarding MS and diet as a significant barrier to the implementation of healthy dietary habits.

I feel as if there are too many conflicting recommendations on what to eat & what not to eat. I wished everyone would get together and formulate one decisive direction for those suffering with MS!

Participants also described that, at times, they struggled to obtain reliable information regarding lifestyle modifications and receive support in their efforts from specialist physicians and other healthcare professionals. In some cases, the information provided was factually incorrect.

My specialist told me that smoking had no effect on MS—which I found somewhat disturbing.

Some participants expressed disappointment and dissatisfaction arising from discussions with their neurologists. They hoped for better support for their efforts and revealed a desire for lifestyle modifications to become central to their discussions alongside medication.

I have found that my MS neurologists and nurses aren't very interested at all in the fact that I have changed my lifestyle significantly by following the OMS programme, which is disappointing as not only do I want them to feel that I am taking the diagnosis seriously but that if I were to be diagnosed again, or if others were, I would want them to tell me about this as a choice as much as the drugs that they can supply.

### 3.3 Physical/psychological barriers

The theme of physical and psychological barriers encompassed personal barriers to adopting or maintaining lifestyle modifications. Three subthemes of the nature and severity of symptoms, no current sense of urgency and motivational barriers were explored.

The type and severity of symptoms stood in the way and affected the ability to implement some lifestyle modifications. Pain and fatigue were understandable barriers to regular physical activity.

I am unable to exercise because of my fatigue and pain.

Conversely, for other participants, current lack of current symptoms hindered the adoption and maintenance of behaviour changes. Current good health seemed to reduce or remove the perceived need to make immediate changes.

I would really like to stick to it, but since I'm generally without symptoms, I find it very hard to stick to anything.

A significant barrier was lack of motivation, with many expressing following through with changes, such as diet, for long periods of time challenging.

I used to follow diet much more, but fell off the bandwagon. I'm slowly getting back into the swing of things, and I really want to find the willpower to keep it up permanently…

Although participants found that the modifications resulted in significant improvements, this was often not enough to maintain motivation to sustain the changes.

I find that when I stick to the MS recovery … I feel so much better. I really struggle to build up enough motivation to stick to it sometimes.

Some participants expressed that uncertainty arising from the conflicting information described in practical challenges led to uncertainty and even fear, and these emotions themselves became barriers to successful adoption of lifestyle changes.

… I understand that many changes need to be undertaken such as regular exercise and eating healthy and regularly. Overall, this is scary and not knowing what to do and what would help is undoubtedly the hardest thing.

At times, there were complex interactions between physical and emotional factors that presented barriers.

I believe that fatigue interferes with my motivation to maintain OMS lifestyle changes. I seem to be in a vicious circle; I am fatigued, so crave sugary foods, which makes me feel fatigued, which makes me crave sugary foods, etc. My lack of motivation to maintain lifestyle changes baffles me and I don't understand why I don't feel inclined to do everything I can to overcome MS.

### 3.4 Enablers of change

The theme of ‘Enablers of change’ described the factors that participants described as having assisted and facilitated the adoption and maintenance of lifestyle modification. Three subthemes were identified: positive attitude, support from others and the use of lifestyle modification resources.

Mind management is a key plank in my MS management—ie manage the negative thought pattern and remain positive. Goal setting and goal achieving are key components in this space—along with a fantastic life coach/mentor.

Participants described that positivity both arose from and facilitated lifestyle modification.

I am very optimistic that I am healing and will be fine and I am trying to follow the diet advice and other advice.

Participants also held strong positive beliefs in spite of adversity. Despite witnessing family members struggle with MS, many participants still had a positive view about their future with the disease and their ability to adopt lifestyle modifications to overcome future obstacles.

My mother had MS, when she was diagnosed, she sat down and became a cripple, I refuse to let this rule my life, with exercise and diet I believe I can overcome it.

Participants identified support from others as a significant enabler of behaviour change. Support from family was crucial.

I might have given up already if my husband wasn't pushing for me to stick to the diet.

Participants frequently described peer support and engagement with OMS resources, provided them with knowledge and encouragement to facilitate positivity and change.

The inspiration and motivation I gathered while attending 2 retreats … has made the major lifestyle changes I have made to my life almost effortless. I was well on course after reading the first book, but the retreats made a huge difference to my understanding of MS, and especially to my attitude towards having MS and dealing with it.

A private facebook page set up by a group after attending an OMS retreat provides very helpful info (from OMS website and various others) and from individual experiences. Also encouragement, compassion and friendship. It is amazing. Have a few in the group who do research and pass this on through this private page.

When I discovered ‘Overcoming Multiple Sclerosis’ I changed my eating habits and eating behaviours. The website has given me a very positive focus.

### 3.5 Experienced outcomes

The theme of ‘Experienced outcomes’ encompassed participants' experiences of the many and varied effects of lifestyle modification on their lives, including the benefits of physical and psychological well‐being. Four subthemes were identified: hope for the future, wellness, developing control and feeling transformed.

Hope was often an unexpected outcome from initiating lifestyle modifications that enabled pwMS to feel stronger and more energized as a result. Participants reported a shift in perspective to a future that was filled with hope.

Luckily I became a vegan about 6 months before my diagnosis so when I was completely at my wits end and almost my life's end I found the… book I was thrilled to find someone speaking my language. I immediately started to follow the regimen … I am so happy and hopeful now for a future that I thought was doomed.

Lifestyle modifications provided participants with a specific pathway towards improving their disease management. This guidance was also critical for participants' hope for their future.

It has given me real hope with a very frightening illness and the lifestyle changes in my life have provided me with a wonderful quality of life and heightened well‐being.

Participants described the development of a sense of control over their health, in contrast to feelings of uncertainty and fear.

I was introduced to OMS last November, I've changed my diet, lost 3.5 stone and feel much better for it, there is more for me to do, but I feel I'm back in charge!

The positivity, described above as an enabler for change, aided in the development of a sense of control and avoidance of feelings of being overwhelmed. This sense of control then further reinforced feelings of positivity, emphasizing mindset as a helpful tool for managing MS‐related challenges.

For me, frame of mind is important. …I tackle my MS from a positive perspective, and refuse to be overwhelmed by it.

Others described improved physical and mental health and general sense of well‐being and quality of life as an outcome of lifestyle modification.

I feel fantastic, better than ever following this lifestyle!!

I believe that the changes I am making … will greatly improve my MS, stop any depression and exponentially increase my outlook on and quality of life.

Some reported significant transformations in their health that affected and subsequently transformed their experiences of daily living, and enabled participants to re‐engage with their normal routine and activities.

Within 2 weeks of not eating meat or dairy, I was no longer having my afternoon sleeps. I would sleep most of the day due to fatigue. Within 3 months I returned to part time work and within 7 months was working fulltime and study. I now work full time as well as having completed my studies and live a very full life, both socially and for work … it is a complete turnaround.

For some, the health outcomes that they had achieved through lifestyle modification were the motivation to achieve challenging physical transformations.

I have lost 60 pounds (thanks to OMS) and participate in distance running events and triathlons. Ironically, MS has led me to being in better shape than ever before.

Alongside changes to physical health and energy levels, participants also described the psychological transformation from dealing with MS via lifestyle behaviours. Participants found that their diagnosis had provided the impetus for initiating major changes within their life, and although they acknowledged the challenges and trials associated with their diagnosis, they adopted lifestyle changes such as meditation and yoga to transform their inner drive.

I wouldn't be as determined an individual as I am if I hadn't been told of my diagnosis or experienced these symptoms … it has been, and probably always will be, far from plain sailing with these symptoms but I can say that I would never have achieved what I have without them. Meditation and yoga have been the key tool to aid me with this.

Also, some participants who had attended the lifestyle modification workshops had transformative experiences.

The MS retreat was transformational in my attitude and behaviour in living with MS.

The OMS retreat changed my life it was amazing and the support group I have now is so wonderful and I have completely embraced the OMS lifestyle.

## 4 DISCUSSION

We qualitatively assessed responses to an open‐ended free‐text question from a survey of a large international cohort of pwMS. We identified four themes that described the experiences of and attitudes to adoption of lifestyle behaviours by pwMS for disease management, some aligning with those reported previously. Themes of challenges and barriers are familiar within the MS literature. Women with MS who held strong desires to initiate changes to their lifestyle reported challenges such as being hindered by difficulties in sustaining motivation, chronic fatigue and uncertainty around recommended levels of exercise, similar to our participants.^30^ Fatigue and mobility issues have been reported as significant barriers to engaging in healthy behaviours in other studies.^31^ Other barriers, such as lack of support and conflicting information from healthcare professionals, have also been similarly reported by partners of pwMS, who reported that healthcare practitioners were not interested in, and even critical of, their partner's participation in lifestyle programmes.^32^

However, while many participants detailed barriers and challenges to implementing lifestyle modification, our study also identified positive attitudes and perspectives towards the management of MS through lifestyle modification that are less commonly reported. These positive perspectives align with those reported in a study of the perspectives of pwMS towards adopting multiple health behaviours, finding that participants were optimistic in their ability to control MS through health behaviours.^31^

The experience of hope in pwMS has been previously reported as hope for a miracle, for a cure or to remain stable and to learn to cope.^33^ Others have experienced hope via developing social interactions and achieving goals^34^ and by undertaking extreme physical challenges alongside others with MS.^35^ Hope can have a positive impact on psychological and social well‐being.^36^ However, hope arising from the implementation of lifestyle modifications and the observed outcomes, as found in our study, is an uncommonly reported finding. Many participants in our study also credited educational resources, specifically a lifestyle workshop or book, with providing a positive outlook and strong hope for a future that previously appeared bleak. The importance of lifestyle modification as a pathway towards hope in pwMS is a substantial finding.

The concept of transformation in chronic illness has been previously researched. Transformation refers to the process by which people living with chronic illness move beyond focussing on the suffering associated with the illness towards the process of realizing the positive and potentially rewarding aspects of living with a chronic condition. One large metasynthesis of explorations of transformation identifies three stages: initial response, embracing the challenge and integrating new ways of being.^37^ Our participants describe similar processes of embracing the challenges and integrating new ways of being. However, the transformations described here appear to be somewhat of an extension to embracing and integrating new ways of being to tangible physical and psychological transformation from the health intervention that they have adopted, adding a further potential layer to the concept of transformation.

### 4.1 Strengths and limitations

We acknowledge the 8‐year time period from data collection to analysis as a limitation to themes possibly not being reflective of current attitudes and perspectives of pwMS. However, it is likely that our findings are still relevant as they have not previously been described in the literature, in particular, the findings related to the positive experiences and outcomes that many pwMS in our cohort reported after lifestyle modification. The open‐ended format of the question as well as the high proportion of nonresponders to the question of interest limit the generalizability of our findings. Both responders and nonresponders may have attitudes and experiences to lifestyle modification that were not expressed. However, the fact that lifestyle modification was volunteered so frequently in an open‐ended question suggests an important, even pivotal role for this issue in the lives of pwMS. The strengths of our study include experiences and attitudes expressed from over 500 pwMS, specifically regarding the adoption of lifestyle behaviours for the management of MS. Future qualitative studies on this topic may consider the use of structured questions.

## 5 CONCLUSIONS

Our study provides novel insights into the impact of lifestyle modification on pwMS, including enablers of behaviour change and the potential experience of positive outcomes such as enhanced hope and transformation. These are aspects of living with MS that could be further explored in future qualitative research focussing specifically on lifestyle modification to more fully explore and understand people's experiences and the contexts that contribute to positive outcomes more in depth. This expanded knowledge base may support evidence‐based disease self‐management in pwMS.

## CONFLICT OF INTERESTS

George A. Jelinek is the author of and receives royalties from Overcoming MS: the evidence‐based 7‐step recovery program, and is the founder of the OMS Charitable Foundation. Sandra L. Neate and George A. Jelinek have previously received remuneration from facilitation of residential workshops.

## AUTHOR CONTRIBUTIONS

*Conceptualization*: Nupur Nag and Sandra L. Neate. *Methodology*: Sandra L. Neate and Nupur Nag. *Investigation*: Nupur Nag and Angela Donald. *Formal analysis*: Angela Donald and Nupur Nag. *Resources*: Sandra L. Neate and Nupur Nag. *Data curation*: Nupur Nag and Angela Donald. *Original draft preparation*: Angela Donald, Nupur Nag, and Sandra L. Neate. *Writing—review and editing*: Nupur Nag, Sandra L. Neate and George A Jelinek. *Visualization*: Nupur Nag and Sandra L. Neate. *Supervision*: Nupur Nag and Sandra L. Neate. *Project administration*: Nupur Nag. *Funding acquisition*: George A. Jelinek. All authors have read and agreed to the published version of the manuscript.

## ETHICS STATEMENT

This study was conducted according to the guidelines of the Declaration of Helsinki, and approved by The University of Melbourne Human Research Ethics Committee, Ethics ID number 1545102. Informed consent was obtained from all subjects involved in the study.

## Data Availability

Data may not be shared due to conditions approved by our institutional ethics committee, in that all data are stored in re‐identifiable form at The University of Melbourne in the form of password‐protected computer databases, with only approved investigators having access. Quotes have been reported in a nonidentifiable manner.
